# Durability Issues and Challenges for Material Advancements in FRP Employed in the Construction Industry

**DOI:** 10.3390/polym10030247

**Published:** 2018-02-28

**Authors:** Mariaenrica Frigione, Mariateresa Lettieri

**Affiliations:** 1 Department of Engineering for Innovation, University of Salento, 73100 Lecce, Italy; 2 Institute of Archaeological Heritage—Monuments and Sites, CNR–IBAM, Prov.le Lecce-Monteroni, 73100 Lecce, Italy; mariateresa.lettieri@cnr.it

**Keywords:** aging, cold-cured epoxy resins, durability, service conditions, weathering, wet lay-up applications

## Abstract

The use of fiber reinforced polymer (FRP) composites for the rehabilitation of buildings or other infrastructure is increasingly becoming an effective and popular solution, being able to overcome some of the drawbacks experienced with traditional interventions and/or traditional materials. The knowledge of long-term performance and of durability behavior of FRP, in terms of their degradation/aging causes and mechanisms taking place in common as well as in harsh environmental conditions, still represents a critical issue for a safe and advantageous implementation of such advanced materials. The research of new and better performing materials in such fields is somewhat limited by practical and economical constrains and, as a matter of fact, is confined to an academic argument.

## 1. Introduction

Fiber reinforced polymers (FRP) are already used in the rehabilitation/strengthening of built infrastructure realized in concrete and masonry, although the lack of fundamental information on their long-term behavior if exposed to different, possibly severe, environments somewhat limits wider implementation of such systems [1].

Their success is due to a variety of different properties, such as high specific strength and specific stiffness, high durability against corrosion, lower weight, ease of installation, and reduced manufacture time. All these latter properties make FRP preferred to traditional construction materials, such as steel and concrete. Furthermore, a wide choice of materials (polymeric resins and fibers) is commercially available: different structures/components can be created from their combination, with tailored anisotropy and geometry able to satisfy the project requirements. Nevertheless, there are several aspects of this relatively new technology that still need further research and development, particularly concerning their durability.

Existing data on the durability of FRPs employed in this specific field are still not organically collected and rationalized. Discrepancies between results obtained by different durability studies have been even observed and possibly attributed to different materials, processing, or conditioning conditions employed (for instance: different times elapsed before the execution of durability tests), being all fundamental information for a complete understanding of the effects of the external environment on properties of materials and for an accurate prediction of their behavior over their lifetime. This uncertainty hampers the enormous potential of composites in the rehabilitation of constructions, since the acceptable lifetime of products employed in this field should be in the order of one hundred years [2].

There is a need to find reasonable tools not only for the prediction of change in the properties of these materials with time when hardened (i.e., cured) in different but realistic thermo-hygrometric conditions, but also for the determination of the remaining service-life of a structure working in widely variable service conditions. Furthermore, implementation of materials, i.e., the development of long-lasting matrices/adhesives for FRP, is compulsory. For both issues, although the efforts of research are rather active worldwide, no conclusive solutions have been identified yet.

## 2. Components for FRP

The performance and durability of FRPs employed in rehabilitation of civil infrastructure mainly depend on the choice of constituent materials to manufacture the FRP, on the process used to manufacture and to apply the composite, on the load regime, and on the type/level of the environmental exposure. FRP composites for such applications are typically composed of continuous fibers (carbon, glass, aramid) embedded in a thermosetting resin matrix (epoxy, vinyl ester, or polyester resins) that holds together the fibers and transfers the load between them. A thermosetting resin (the same composing the matrix of FRP or a different one) is also employed to act as adhesive between the FRP and the (concrete/masonry) substrate. The chemical nature of the matrix/adhesive for the composite as well as the conditions used to set and harden it will have a decisive influence on the performance and behavior of the FRP and on the effectiveness of the whole intervention.

**Figure 1:**
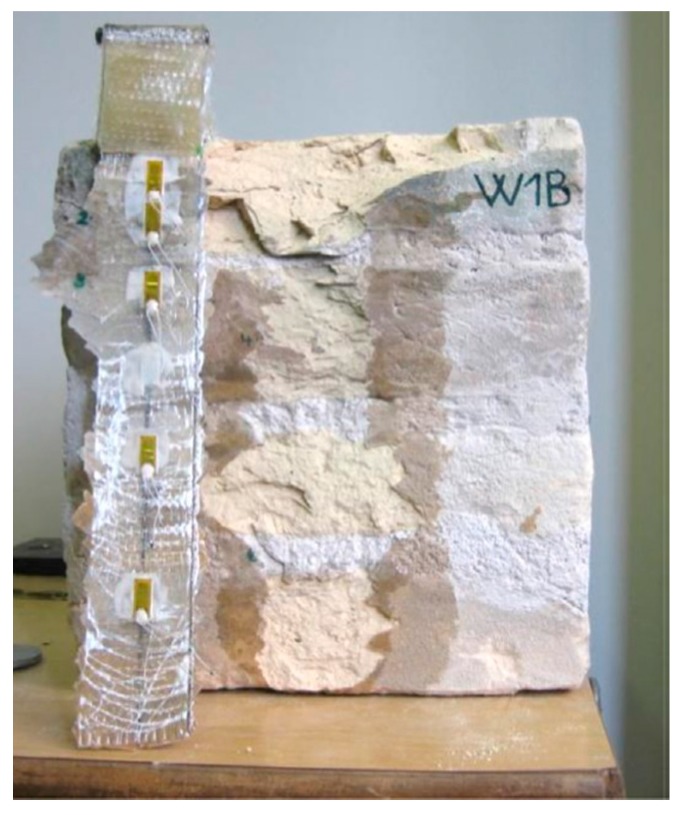
Typical delamination failure of GFRP strip-to-historic-brickwork joint [3]. Reprinted from Composite Structures, 140, Capozucca R., Ricci V., Bond of GFRP strips on modern and historic brickwork masonry, 540–555, Copyright (2016), with permission from Elsevier.

The behavior and integrity of an element reinforced by FRP depend not only on the properties of the individual materials, but also on the performance of the FRP–adhesive and adhesive–substrate interface bonds. Therefore, the reliability of the rehabilitation intervention using externally bonded FRP materials depends to a large extent on the bond between the reinforcement and the substrate and, therefore, on the ability to transfer the stresses at the interface. As an example, experimental tests have proved that composite materials bonded to historic brick joints lose their capacity by brittle delamination failure [3] (Figure 1).

FRP can be applied following two different procedures: (a) the precured FRP prepregs are adhesively bonded as prefabricated elements to the concrete (or masonry) substrate; (b) the composite is applied through a “wet lay-up” of fabrics directly onto the substrate.

In the first case, the application of prefabricated (often pultruded) laminates ensures the use of precured materials, produced in factories through industrially controlled processes, thus achieving a high level of uniformity in the final product that will display high properties. On the negative side, prefabricated FRP elements are less flexible and are not adaptable for unpredicted configurations that can be found in field applications (i.e., the confinement of cylindrical concrete columns, the strengthening of arches and vaults in masonry constructions). Moreover, the application of a precured FRP element to a concrete/masonry substrate is carried out by means of a thermosetting adhesive applied and hardened on site. This, therefore, implies the introduction of an adhesive interphase between the already cured FRP and the substrate [4].

**Figure 2:**
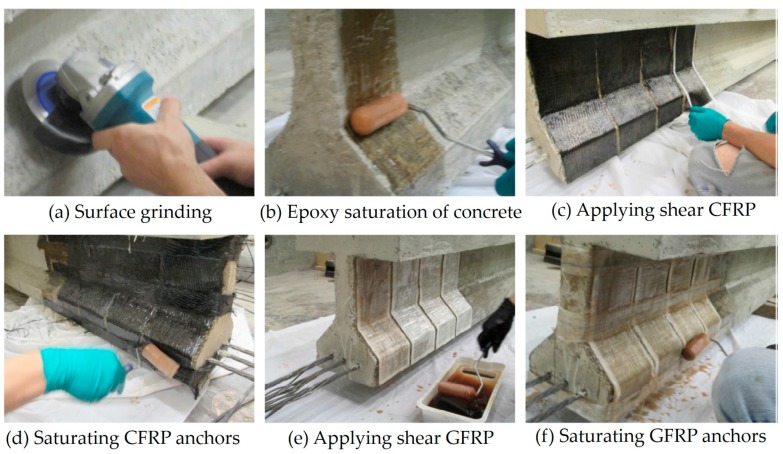
Example of application of CFRP (Carbon Fiber Reinforced Polymer) and GFRP (Glass Fiber Reinforced Polymer) by wet lay-up technique [7]. Reprinted from Construction and Building Materials, 148, Shaw I., Andrawes B., Repair of damaged end regions of PC beams using externally bonded FRP shear reinforcement, 184–194, Copyright (2017), with permission from Elsevier.

The use of the wet lay-up technique (i.e., the FRP is applied and formed in situ (Figure 2)) provides enormous flexibility, since the pre-impregnated fabrics can closely follow the geometrical configuration of the structure to be rehabilitated. Moreover, the bond between the FRP and the substrate is guaranteed by an adhesive resin that is very similar to the matrix of the composite, i.e., it is able to form a continuum between the FRP and the substrate. The lack of a careful control of the curing process, however, leads to a significantly higher level of variation in the final performance of the intervention.

In both the described techniques, the weakest link is represented by cold-cured thermosetting resins, often epoxy, used as adhesive in the first case and as matrix/adhesive in the second. While the resin is responsible for the overall integrity of the rehabilitated structure, since it must assure an effective stress transfer among the structure and the FRP reinforcement, it can undergo both chemical and physical degradation by environmental actions and mechanical stresses.

Interactions can take place between polymer adhesives and concrete substrates [5], affecting the local mechanical properties of the adhesive along the interface and reducing its resistance to environmental effects [6].

The properties and durability of the FRP–substrate bond can be enhanced by surface modification through mechanical treatments [7,8] or functionalization with coupling agents (usually silanes), the latter being able to change the interfacial hydrogen bonds to stronger covalent ones [9,10,11,12].

### 2.1. Cold-Cured Thermosetting (Epoxy) Resins

Among the polymers employed in this field, epoxy resins are without a doubt the most used due to their excellent properties. They can be formulated into low viscosity systems which “cure” (i.e., form cross-links throughout the structure and, as a consequence, harden) at room temperature with a minimal shrinkage; when correctly formulated and cured, they exhibit a good combination of mechanical properties and chemical resistance towards environmental agents; compared to other resins, cured epoxy systems are known to have excellent adhesion to a broad range of substrates and reinforcing materials. For the strengthening/repairing applications through FRPs, epoxies are frequently preferred to both vinyl ester and unsaturated polyester resins, both characterized by an excessive shrinkage during curing, with the possible formation of micro-cracks or micro-gels, resulting in micro inhomogeneity and incomplete polymerization [13]. Unsaturated polyester resins, in addition, display high susceptibility to moisture and low bonding efficiency in damp or wet conditions and when exposed to alkaline environments.

The polymerization (curing) reactions of an epoxy (part A of the system), giving rise to a rigid network-type structure, occurs in the presence of a suitable curing agent (hardener, part B of the system) and is favored by heat/radiations, depending on the ingredients (Part A and B) and on the curing mechanism. In particular, the kind and amount of the hardener are selected on the basis of the resin and on the available curing conditions and have both an appreciable influence on the final performance of the cured epoxy.

For economic and practical reasons, the resins used as matrix and/or adhesive for FRP components employed for the rehabilitation of constructions are “cold-cured” types, typically based on bis-phenolic epoxies, cured at ambient temperatures on site with the addition of aliphatic amines [14]. Unlike the epoxy resins employed as matrices for FRP and adhesives in the much more demanding aeronautical/aerospace or automotive industries (that are typically cured employing curing cycles characterized by very high temperatures with the addition of curing agents, i.e., aromatic amines or anhydrides, active only at these temperatures), epoxy resins used in the construction industry, where large surfaces must be strengthened by an FRP often formed on field (i.e., in situ), are cross-linked without the possibility to effectively control and keep constant the manufacturing procedures as well as the (outdoor) conditions for the processing (hardening) of the adhesive/matrix resin. Providing any kind of heat source over the large areas required for the described applications, in fact, is very difficult and prohibitively expensive.

The main consequences of a cure at ambient (often not-constant and uncontrolled) temperatures of epoxy adhesives are: (i) long curing times (in the order of weeks) are necessary to achieve sufficient mechanical properties, the lower the curing temperature, the longer the curing time; (ii) the curing (cross-linking) reactions taking place at ambient temperatures are often not completed due to kinetic restraints; (iii) a moderate glass transition temperature (*T_g_*), in practice never greater than 65–70 °C, is attainable by these systems, particularly if the curing of the resin occurs at low winter temperatures [15,16,17,18,19,20,21]. In addition, the absorption of external water (for example, as atmospheric moisture or rain) produces a decrease in the initial *T_g_* of the resin, with consequent negative effects on mechanical and adhesive properties [22,23].

When the *T_g_* is approached and surpassed by an even mild external temperature, the adhesion between the FRP element and the substrate (concrete/masonry) is likely to be reduced. Furthermore, the exposure to moderate temperatures (above the *T_g_*) of not fully cross-linked thermoset polymers can promote their post-cure. The post-cure is usually reflected in increment of *T_g_*, strength, and stiffness of the resin [21,24,25]. However, *T_g_* values never exceeding 75 °C are generally found for an epoxy/aliphatic amine couple, even if the cross-linking of the resin has been completed through a post-cure procedure [20]. These systems, therefore, operate in a non-equilibrium state, with the properties evolving in time and as a consequence of the variable external conditions.

### 2.2. Fibers and Configuration of FRP

Referring to the fibers, carbon is the most commonly fiber used in FRP systems for rehabilitation applications where exposure to aggressive environments is expected. Carbon fibers are, in fact, considered to be inert to most environments that can be found in civil infrastructure applications. The less expansive glass fibers, on the other hand, are more susceptible to harsh environments, especially moisture/alkaline ones, the latter producing loss in toughness, strength, and embrittlement. Nevertheless, the durability of glass fibers upon exposure to typical outdoor applications is still satisfactory, especially if they are suitably protected against harsh agents by tailored sizing coatings, also acting as a bond-enhancement. The durability of glass fiber reinforced polymers (GFRP) can be even improved by using hybrid glass-carbon fabrics.

Other parameters have a crucial influence on the behavior of the FRP-rehabilitated structures, i.e., the number of composite plies, the direction and disposition of fibers in each ply, and their weave pattern. The configuration of FRP components, in fact, must be properly designed taking into account the complex system of forces to which the structure rehabilitated with the FRPs will be subjected over its lifetime.

## 3. Durability of FRPs in Common or Harsh Environments

The environmental conditions to which FRPs for rehabilitation of structures are more frequently exposed during their service life are neither constant nor predictable and depend on several parameters, such as: the latitude and the altitude of the site, the season, the distance from sea, and the local weather. Environmental factors can severely affect the performance in service of each element composing the FRP and of the whole FRP, even after a short time from its installation, due to specific processes, either reversible or permanent, taking place between the external agents and the materials composing the FRP. In particular, the role of the matrix/adhesive on the behavior of the FRP system is crucial. Due to the peculiarities of cold-cured epoxy resins, in fact, the environmental conditions most frequently encountered in civil infrastructures may severely affect the performance of wet lay-up type FRP and, more generally, the integrity of FRP-to-FRP and FRP-to-substrate bonds.

The service conditions characteristic of the common outdoor applications include atmospheric humidity, rain, solar (UV) radiations, large variations in temperature, freeze-thaw regimes, acid rain, sea-water, deicing chemicals, alkaline environment when in the proximity of Portland cement concrete, and sustained loads. Polymer composites can be also accidentally exposed to extreme environments, such as fire, earthquake, and explosive blasts.

The presence of humidity in the air, either in the form of moisture or actual water through rain, is probably the most harmful environment that can be encountered by matrices and adhesives employed in FRPs for civil engineering applications. Epoxy resins are able to absorb substantial amounts of water due to the presence of polar groups able to attract water molecules. The ingress of moisture over time is particularly significant if the polymer is permanently immersed in water, or salt or alkaline solutions, or if it is exposed to deicing salt solutions. An excessive penetration of water is generally considered harmful since it leads to a reduction in stiffness (even halved) and strength of the resin, with a consequent remarkable reduction of the *T_g_* of the resin and a marked decrease of load-bearing capacity, due to plasticization effects [8,15,16,26].

When the service temperature approaches the *T_g_* of the resin, a dramatic decrease (up to 70%) of the (mechanical, adhesive) properties of the cold-cured resin occurs: approaching the *T_g_*, the behavior of the resin drastically changes from that of a solid adhesive, able to effectively bond two different materials, to that of a soft material, unable to guarantee the stress transfer between the same materials. Even a moderate service temperature, therefore, is able to appreciably reduce the adhesion strength to concrete, i.e., by over 80% at 50 °C, as well as the fatigue resistance [27]. As the temperature and/or the exposure time increase, the mechanism of failure occurring in the samples changed, from predominantly concrete failure to mixed failure of epoxy and at the interface [28]; this transition point changes with temperature, that is, increasing the temperature, the transition occurs at higher values of pull-out force [29]. The service temperatures encountered in practice by these systems may be close or even higher than their *T_g_*; even if the air temperature measured in Mediterranean areas usually does not exceed 40–45 °C even in summer, the temperature of a surface irradiated by sun can be appreciably higher, i.e., even greater than the *T_g_* of the matrix/adhesive resin.

**Figure 3:**
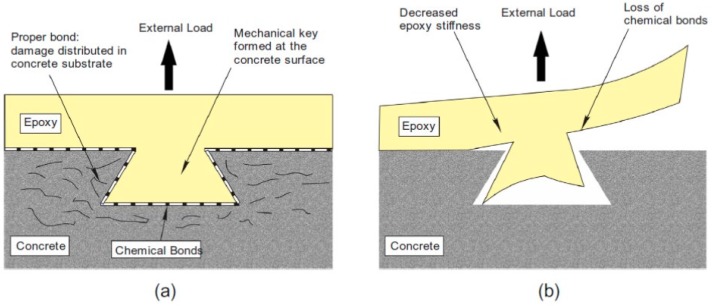
Failure mode of epoxy–concrete bond: (**a**) in dry ambient conditions; (**b**) following exposure to moisture [23]. Reprinted from Construction and Building Materials, 96, Blackburn B.P., Tatar J., Douglas E.P., Hamilton H.R., Effects of hygrothermal conditioning on epoxy adhesives used in FRP composites, 679–689, Copyright (2015), with permission from Elsevier.

The detrimental effects of water or moisture and of moderate temperatures on the performance of a cold-cured epoxy resin are also reproduced on its behavior as an adhesive [30]. It was found, in fact, that both agents are able to appreciably reduce the bond properties between an epoxy adhesive and concrete elements [31] (Figure 3).

However, satisfactory durability properties of the adhesive/composite do not guarantee proper FRP-to-concrete bond durability [32]. The bond between FRP and concrete is established through a combination of mechanical interlocking and chemical bonding. The durability of the bond can be affected not only by the variation of the epoxy resins (via plasticization), but also by interferences in the bond-forming mechanisms. In particular, the presence of water molecules at the interface is able to disturb the interfacial hydrogen bonds between the epoxy and the concrete substrate [10,33].

As a general conclusion, when using the cold-cured resins, due to their moderate *T_g_*, attention must be given to the site temperature: the (maximum) environmental temperature under working conditions should be at least 20 °C below the expected glass transition temperature of the resin [14].

**Figure 4:**
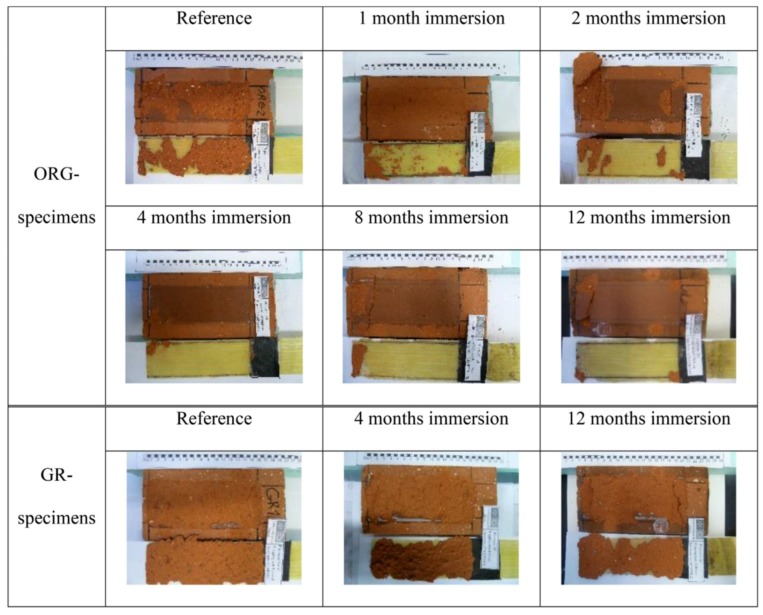
Failure mode changes with water immersion [8]; specimens prepared by wet layup procedure on grinded surfaces (GR) or without mechanical surface treatment (OGR). Reprinted from Composites Part B, 87, Maljaee H., Ghiassi B., Lourenço P.B., Oliveira D.V., Moisture-induced degradation of interfacial bond in FRP-strengthened masonry, 47–58, Copyright (2015), with permission from Elsevier.

Referring to the influence of water/moisture on the performance of FRP, apart from the kind of matrix resin and fibers used (carbon vs. glass), it mainly depends on the configuration of fabrics and on the direction of application of the load. In the case of in-plane tensile tests performed on unidirectional single ply wet lay-up FRP, for instance, only a negligible influence from the presence of water was found [18]. On the other hand, in laminates composed by several plies, the presence of water at the interface between the adjacent layers is likely to be severely harmful. Since the matrix resin is also responsible for the adhesion between plies, greater reductions in tensile strength are found for thicker specimens, i.e., composed by a large number of plies. Similarly, the presence of water/moisture at the adhesive/fibers/substrate interfaces is detrimental when the FRP is applied to a concrete/masonry substrate [34,35,36,37]. The failure mode, initially cohesive with fracture inside the substrate, changed to cohesive-adhesive then to adhesive failure at longer exposure time (Figure 4).

Severe degradation in FRP properties occurs upon exposure to freeze-thaw regimes due to the stiffening and embrittlement of the matrix, with possible formation of micro-cracks. Fiber-matrix debonding and a local loss of adhesion strength towards substrate may take place due to the difference in coefficients of thermal expansion. Reductions in tensile strength and interlaminar fracture toughness are generally observed after repeated freeze-thaw cycles [38]. The loss in strength is even more severe when the thaw regime is performed in saline environments. Seawater, deicing salts, alkaline, and acid solutions are particularly harmful for AFRP (aramid fiber reinforced polymers) and GFRP, producing damage in both at the fiber–resin interface and the degradation of the glass fibers in GFRP [39]. The tensile properties of CFRP (carbon fiber reinforced polymers) are scarcely affected by immersion in alkaline and acid solutions, while their flexural and interlaminar characteristics are affected by both chemicals. Weathering under marine environment produces a reduction in compressive strength mainly due to bond degradation between CFRP and concrete with a greater effect in short term rather than in the long-term exposure [40].

Researches devoted to investigation of the effects of long-term and field exposure on FRPs are still limited [41,42,43] and only a few studies focused on the effects of outdoor exposure on epoxy matrices/adhesives. It is generally found that natural weathering (with UV radiations and thermo-hygrometric variations) caused changes in both physical and mechanical properties, whose effects could be partly reversible, such as plasticization, but also non-reversible, such as hydrolysis and post-curing [44,45,46,47]. The adhesive bond strength as well as the performance of the whole FRP would be consequently affected by the same weathering parameters, with degradation dominated by resin and interface deterioration mechanisms [48]. Nevertheless, the overall performance of the FRP have proven to be generally satisfactory when relatively mild climates are experienced [49,50].

Apart from the degradation of bond performance, as in the previously discussed case of exposure to elevated temperatures, polymer composites display a huge vulnerability against fire since resins are organic materials mainly composed of carbon and hydrogen, both highly flammable. The performance and behavior of a repaired concrete beam under fire depend also on the type of cracks, repaired using epoxy, and on the extent of repair [51]. In addition, severe health hazards derived from polymers and composites in a fire accident is generated from the toxic combustion products created during burning of materials.

In order to reduce the fire hazards in FRP, therefore, it is recommended to: (i) provide thermal protection for structures on site; (ii) to introduce flame retardant agents (i.e., halogen based) into the resin formulations; (iii) or to apply a protective intumescent coating on the surface of the manufactured composite [14].

Although fillers like aluminum or magnesium hydroxides are among the cheapest and most effective fire-retardant agents, they significantly deteriorate the mechanical and electrical properties and the rheology of the pristine resin. Halogen additives, on the other hand, are among the most effective agents for reducing the rate of heat release of phenolic, epoxy, or bismaleimide resins; however, a high loading of such additives is often required, with associated cost, processability, and property penalties.

When phosphorous-based flame retardants are purely blended with epoxy resins, they, not being chemically bonded to the network, can migrate toward the surface of components before cross-linking, reducing the glass transition temperature of the cured resin by acting as plasticizer [52]. On the other hand, reactive organo-phosphorus compounds show more excellent flame-retardant efficiency: the reactive flame retardants can be directly incorporated into the backbone of the epoxy network, either as a part of the curing agent or the epoxy itself, effectively exploiting their capability.

## 4. Development of Improved Materials

The use of nano-structured polymers as matrix/adhesive for FRP is expected to become a realistic alternative to traditional polymeric products in the civil engineering field due to their superior properties and greater durability against moisture, temperatures, hash environments, and fire. Nano-structured polymers are typically produced as nano-composites, based on preformed nano-sized inorganic particles (clay [53,54,55,56], carbon nano-tube [57,58], graphene [59], inorganic nanoparticles [60,61,62,63] such as SiO_2_ [64], ZnO [65,66], Al_2_O_3_ [67], and TiO_2_ [68]).

**Figure 5:**
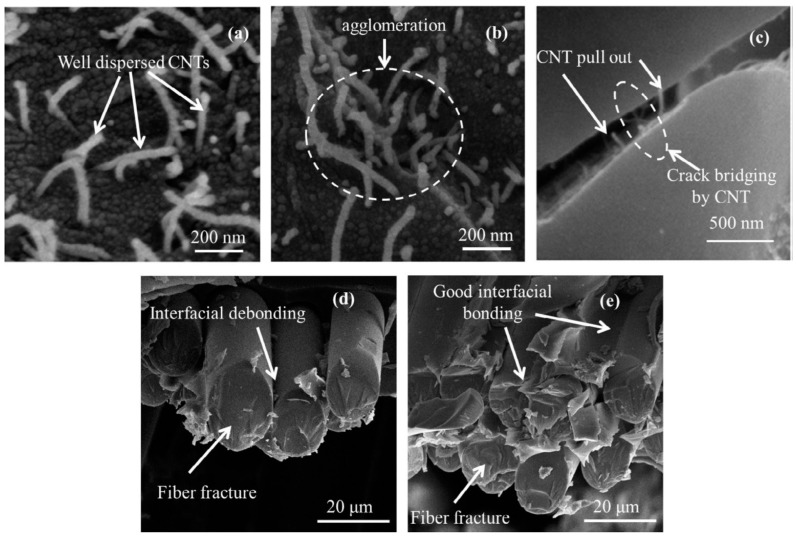
Dispersion of (**a**) 0.1%; (**b**) 0.5% multi walled carbon nanotubes (MWCNT) in glass/epoxy (GE) composite; (**c**) CNT pull out and crack bridging by CNT in 0.1% MWCNT–GE composite after room temperature test; glass fiber bundle (**d**) GE and (**e**) 0.1% MWCNT–GE composite [58]. Reprinted from Composites Part A, 84, Rathore D.K., Prusty R.K., Kumar D.S., Ray B.C., Mechanical performance of CNT-filled glass fiber/epoxy composite in in-situ elevated temperature environments emphasizing the role of CNT content, 364–376, Copyright (2016), with permission from Elsevier.

Nanoparticles can be used to improve the mechanical properties of the matrix, which comprises both resin and nano-sized filler [69]. The presence of well dispersed nanoparticles in the matrix enhances the phase adhesion [53,56,70] (Figure 5); constraint to polymer chain movement [55,66,71] is also observed, thus, higher *T_g_* values are found [55,56,72,73].

The main difficulty experienced in the production of such nanocomposites is the non-uniform dispersion of the nanofiller into the resin matrix [60]. Mechanical methods (e.g., ultra-sonication) are usually used to properly mix the components because of their cost-efficient, eco-friendly, and single-step application. A chemical approach is required where the mechanical action can alter the structure of the nanomaterials, diminishing their performance [62,74].

Another widely practiced way, especially to improve impact properties of the composite and reduce the crack propagation [69], is the production of composites where nanofillers are grafted onto the traditional fibers [75,76,77]. Coating (dipping or spraying), chemical vapor or electrophoretic deposition, and addition of chemical binding agents are the most used methods to modify the fibers [62,78].

**Figure 6:**
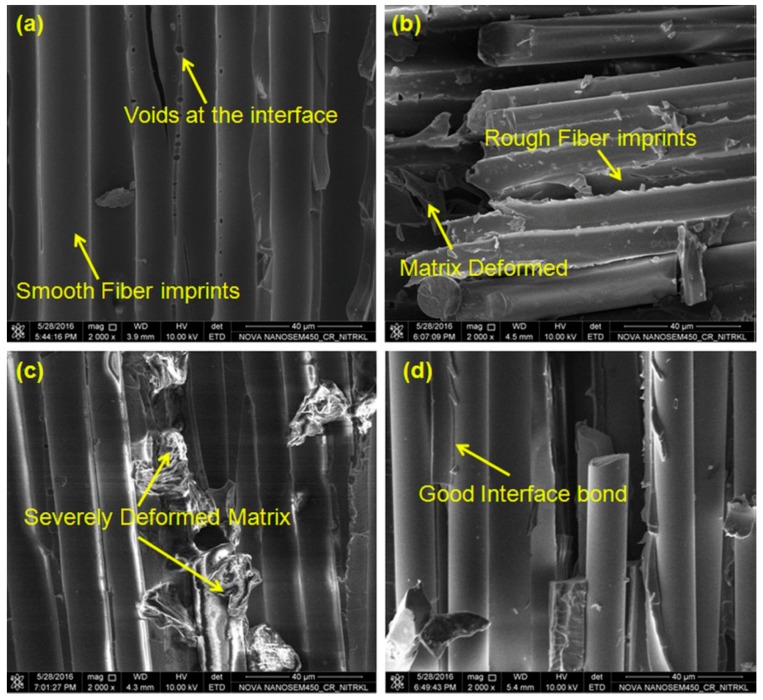
Field Emission scanning Electron Microscopy (FESEM) images of fractured surface of hydrothermally conditioned samples having different wt % of nano-Al_2_O_3_: (**a**) 0.0; (**b**) 0.1; (**c**) 0.3; (**d**) 0.7 [67]. Reprinted by permission from Springer Nature: Springer, Polymer Bulletin 74: 4175–4194, Water absorption, residual mechanical and thermal properties of hydrothermally conditioned nano-Al_2_O_3_ enhanced glass fiber reinforced polymer composites, Ramesh Kumar Nayak, Bankim Chandra Ray, Copyright (2017).

Besides improved mechanical, thermal, and electrical properties, changes in resistance to the damage [53] and lesser sensitivity to the environmental agents [79,80] have been found for the nanocomposites. However, the type, amount, and distribution of nanoparticles determine whether the matrix/composite properties improve or deteriorate [70,73]. The presence of hydrophilic nanoparticles (e.g., nanoclays) may produce increased water absorption, swelling, and hydrolysis [54,67,72,73,81], but a good particle’s dispersion is able to provide a barrier to gas/liquid [80]. Interactions between matrix and nanoparticles or agglomeration may reduce the *T_g_* [58,59]. High addition of nanoparticles (as a filler) in the composite can lead to crack formation, voids, and other defects, leading to degradable mechanical properties [79] (Figure 6). Poorly dispersed nanocomposites may also have degraded mechanical properties [56].

The nanoparticles in FRPs are also able to increase the resistance under fire conditions [63,82,83,84,85,86]. In addition, also the post-fire residual strength can be improved in nanocomposites [56]. A better fire resistance can be obtained by incorporating the nanofiller into the matrix [82] or modifying the material surface with a flame-retardant nanocoating [87]. In both cases, the fire-retardancy originates from a dense char layer formed during combustion, which, acting as a barrier against heat propagation and gas diffusion, protect the underlying structure [82,87,88]. The quality of the protective surface layer formed during combustion, and then the nanocomposite flame retardancy, are strongly affected by the dispersion of nanofillers [82,88]. Although the addition of nanofillers can prevent flash-over and the spreading of flame [84], self-extinguishing is not guaranteed, therefore, the addition of conventional flame retardants is strongly suggested to fulfill safety requirements [56,82].

More recently, plant-based fibers and recycled materials have been studied as both fibers and fillers for reinforced composites [68,89,90,91,92,93,94]. The growing attention to environmental protection and sustainable development make them a suitable option to replace the traditional products. However, although good thermal and mechanical properties can be achieved, interfacial adhesion between fiber and matrix is still the key issue in terms of final performance, limiting the application of these composites.

The efforts of academic and industrial research in this field must be mainly devoted to the development of more durable thermosetting matrix/adhesive resins at affordable costs, also able to achieve a stable thermodynamic state after short curing times in different thermo-hygrometric conditions, with a consequent improvement in the long-term performance of FRPs. The formulation strategy of these systems, then, must be aimed at increasing the *T_g_* and the elastic modulus in the rubbery region of the resin as well as at improving their performance under different environmental regimes, irrespective of the curing conditions [95].

At the present time, however, the production processes of such nano-structured polymers are still too complicated and expensive to be conveniently applied in the construction industry.

## 5. Conclusions

Due to the heavy concerns about the durability of FRP materials intended for rehabilitation of constructions, in common as well as in harsh environments, research should be forced to the development of new epoxy resins still able to set and cure at ordinary temperatures and humidity levels but displaying much greater *T_g_* values, lower curing times, and less durability concerns than those commercially available at the present time. Despite the described drawbacks, these resins still represent a viable solution to assemble and/or to apply FRP repairing elements. These latter, in turn, display several advantages over traditional materials in terms of high strength-to-weight and stiffness-to-weight ratios, great versatility, shorter times for the interventions, and, consequently, of activities interruption, with a consequent reduction of the overall costs. Maintenance operations are also cut when polymer composites are applied in substitution of traditional construction materials. The collaboration between experts possessing different scientific background and expertise is, therefore, greatly encouraged for a stronger, deeper comprehension of durability phenomena and a faster successful identification of practicable solutions.
